# The Olive Oil-Based Lipid Clinoleic Blocks Leukocyte Recruitment and Improves Survival during Systemic Inflammation: A Comparative In Vivo Study of Different Parenteral Lipid Emulsions

**DOI:** 10.1155/2015/757059

**Published:** 2015-02-12

**Authors:** Kirsten Buschmann, Johannes Poeschl, Natascha Braach, Hannes Hudalla, Navina Kuss, David Frommhold

**Affiliations:** Clinic of Neonatology, Department of Pediatrics, University of Heidelberg, 69120 Heidelberg, Germany

## Abstract

Although fish oil-based and olive oil-based lipid emulsions have been shown to exert anti-inflammatory functions, the immunomodulating properties of lipids are still controversial. Therefore, we investigated the anti-inflammatory effect of three different parenterally administered lipid emulsions in vivo: olive oil-based Clinoleic, fish oil-based Smoflipid, and soybean oil-based Lipofundin. We observed leukocyte recruitment in inflamed murine cremaster muscle using intravital microscopy and survival in a murine model of LPS-induced systemic inflammation and analyzed expression of leukocyte and endothelial adhesion molecules. Olive oil-based Clinoleic and fish oil-based Smoflipid profoundly inhibited leukocyte adhesion compared to Lipofundin during LPS-induced inflammation of the murine cremaster muscle. In the trauma model of cremaster muscle inflammation, Lipofundin was the only lipid emulsion that even augmented leukocyte adhesion. In contrast to Smoflipid and Lipofundin, Clinoleic effectively blocked leukocyte recruitment and increased survival during lethal endotoxemia. Flow chamber experiments and analysis of adhesion molecule expression suggest that both endothelial and leukocyte driven mechanisms might contribute to anti-inflammatory effects of Clinoleic. We conclude that the anti-inflammatory properties of Clinoleic are superior to those of Smoflipid and Lipofundin even during systemic inflammation. Thus, these results should stimulate further studies investigating parenteral lipids as an anti-inflammatory strategy in critically ill patients.

## 1. Introduction

Parenteral nutrition is crucially important in critically ill patients [1, 2]. However, numerous studies reported complications during parenteral nutrition, like parenteral nutrition associated liver disease [3] or detrimental effects of parenteral lipids on survival and inflammatory response during sepsis [4, 5]. This may also be due to lipid-induced decrease of neutrophil function and cytokine release in septic patients [6, 7]. Therefore, strategies to avoid the negative consequences of intravenously administered lipids were needed [4, 8, 9].

In recent years, the composition of lipids was mainly based on soybeans which contain high amounts of omega-6-polyunsaturated fatty acids. During the beginning of parenteral nutrition, Intralipid which only contains soybean-based long-chain-triglycerides (LCT) was frequently used. Omega-6 fatty acids belong to the family of polyunsaturated fatty acids (PUFA) and are precursors of eicosanoids. Eicosanoids act as immunomodulators, serve as signaling molecules, and contribute to inflammatory conditions [10]. In this context, they promote leukocyte recruitment by increased production of proinflammatory cytokines. On the other hand, they negatively affect lymphocyte proliferation, thereby causing an immunosuppressive effect [11, 12]. One important aspect of the observed effects is the change of cell membrane fluidity by parenterally administered fatty acids [13].

In order to attenuate these serious side effects of PUFA, new lipid emulsions were formulated for parenteral nutrition. Lipofundin is one alternative that substituted 50% of LCT with medium-chain-triglycerides (MCT) that are metabolized more rapidly than LCT, thereby displaying less immunosuppressive properties and exerting better effects on membrane function [14]. One study that compared the respiratory burst of human neutrophils found a reduced effect with Lipofundin when compared to other lipid emulsions [15]. In contrast to Lipofundin and Intralipid, olive oil-based lipids were shown to have a protective effect against LPS-induced inflammation [16]. Olive oils contain high amounts of monounsaturated fatty acids (MUFA) and are known to show less sensitivity to peroxidation when compared to PUFA. However, there is an ongoing discussion about beneficial properties of fish oil-based lipids (i.e., Smoflipid) when compared with predominant olive oil-based lipids (i.e., Clinoleic) [17, 18].

Smoflipid contains fish oil, which is rich in omega-3 fatty acids and is able to inhibit the production of proinflammatory cytokines via activation of peroxisome proliferator-activated receptor (PPAR) and interaction with NFkB [19, 20]. The beneficial effects are at least in part attributed to a favorable ratio of omega-6: omega-3 fatty acids and the balanced mixture of different lipid ingredients (LCT, MCT, and olive oil) [18].

In addition, there are still conflicting results about how parenterally administered lipids might interfere with leukocyte recruitment which is known to be a sensitive indicator of inflammation. Leukocyte recruitment into inflamed tissue follows a well-defined cascade of events beginning with the capture of free flowing leukocytes to the vessel wall followed by leukocyte rolling (mediated by selectins and their ligands) triggering the activation of *β*_2_-integrins (i.e., LFA1, MAC1) by chemokines and their receptors (i.e., CXCR2). Once activated, leukocyte's integrins can bind to their respective endothelial receptors, like ICAM-1 or VCAM-1. This in turn leads to firm leukocyte arrest on the endothelium and finally the leukocyte transmigration into the tissue [21, 22].

Based on the above-mentioned controversies about lipid-induced immunomodulation, we now aimed to compare anti-inflammatory effects of Lipofundin, Smoflipid, and Clinoleic in vitro and in vivo with special regard to leukocyte recruitment during local and systemic inflammation.

## 2. Materials and Methods

### 2.1. Animals

C57bl/6 wild type (WT) mice were provided by Charles River (Sulzfeld, Germany). All mice were maintained as breeding colonies at the Central Animal Facility of the University of Heidelberg, Germany. For intravital microscopy experiments, mice were at least 8 weeks of age. The animal experiments were approved by the Animal Care and Use Committee of the Regierungspräsidium Karlsruhe, Germany (Az 35-9185.81/G-3/13).

### 2.2. Lipid Emulsions

Clinoleic (Fresenius Kabi, Bad Homburg, Germany) contains 80% olive oil and 20% soybean oil (LCT). Smoflipid (Fresenius Kabi, Bad Homburg, Germany) consists of 30% soybean oil (LCT), 30% MCT, 25% olive oil, and 15% fish oil (rich in Omega 3 fatty acids). Lipofundin (Braun, Melsungen, Germany) consists of 50% soybean oil (LCT) and 50% MCT. The lipid emulsions were administered as an intravenous bolus injection at 1 g/kg for the intravital microscopic experiments and at 2 g/kg 30 min, 8 h, and 24 h after LPS during LPS-induced endotoxemia. Lipid emulsions were incubated during the in vitro experiments as indicated.

### 2.3. Intravital Microscopy

Mice were prepared for intravital microscopy, as reported recently [23]. Briefly, after intraperitoneal (i.p.) injection of ketamine (125 mg/kg body weight, Ketalar; Parke-Davis, Morris Plains, NJ, USA) and xylazine (12.5 mg/kg body weight; Phoenix Scientific Inc., St. Joseph, MO, USA) mice were placed on a heating pad to maintain body temperature. Intravital microscopy was conducted on an upright microscope (Leica; Wetzlar, Germany) with a saline immersion objective (SW40/0.75 numerical aperture, Zeiss, Jena, Germany). Mice were intubated and the left carotid artery was cannulated for blood sampling and the right jugular vein for lipid administration. The lipid emulsions were administered with a dose of 1 g/kg as an intravenous bolus injection. Blood levels of cholesterol, triglycerides, and liver enzymes were measured after the respective experiments in the core laboratory facility of the University Hospital Heidelberg (Analysezentrum, Heidelberg, Germany).

### 2.4. Cremaster Muscle Preparation

The surgical preparation (trauma-induced inflammation) of the cremaster muscle was conducted as described previously [24]. Briefly, the scrotum was opened and the cremaster muscle was exteriorized. After longitudinal incision and spreading of the muscle over a cover glass, the epididymis and testis were mobilized and pinned aside leading to full microscopic access to the cremaster muscle microcirculation. Cremaster muscle venules were recorded via CCD camera (CF8/1, Kappa, Gleichen, Germany) on a Panasonic S-VHS recorder. S-VHS tapes were digitized using a DVD maker (Typhoon, Schalksmuehle, Germany). The cremaster muscle was superfused with thermocontrolled (35°C) bicarbonate-buffered saline. Postcapillary venules under observation were recorded before and during lipid administration and ranged from 20 to 40 *μ*m in diameter. Systemic blood samples (10 *μ*L) were taken and assessed for white blood cell count after staining with Turk's solution 1 : 10 (Merck, Darmstadt, Germany) using a hemocytometer. Microvascular parameters (venular diameter, venular vessel segment length) were measured using an image processing system [25]. Venular centerline red blood cell velocity was measured during the experiment via a dual photodiode and a digital on-line cross-correlation program (CircuSoft Instrumentation, Hockessin, DE, USA). An empirical factor of 0.625 was used to convert centerline velocities to mean blood flow velocities [26]. Wall shear rates (*γ*_*w*_) were estimated as 4.9 (8*v*_*b*_/*d*), where *v*_*b*_ is mean blood flow velocity and *d* is the diameter of the vessel [27, 28]. The number of adherent leukocytes (firm adhesion for >30 s) was assessed as adherent cells per mm^2^ vessel surface area as reported previously [24]. Rolling leukocyte flux fraction was defined as the percentage of rolling leukocytes to all leukocytes passing the same vessel in 1 minute [29]. In certain experiments mice were injected with 50 ng LPS (*Escherichia coli*; serotype 055:B5 Sigma, Taufkirchen, Germany) intrascrotally (LPS-induced inflammation).

### 2.5. Whole Mount Histology

To differentially count transmigrated leukocytes, cremaster muscle-whole mounts were prepared as described before [30]. Briefly, while the cremaster muscle was still mounted on the stage for intravital microscopy, the tissue was fixed with 4% paraformaldehyde in 0.1 M phosphate buffer (pH 7.4). The cremaster muscle was removed and mounted flat on a superFrost glass slide (Menzel, Braunschweig, Germany), air dried for 5–10 min, and fixed in 4% paraformaldehyde in 0.1 M phosphate buffer (pH 7.4) for 24 h at 4°C. After fixation, the tissue was washed three times in 0.1 M phosphate buffer with 5% ethanol, stained with Giemsa (Sigma, Taufkirchen, Germany) at room temperature for 5 min, and differentiated in 0.01% acetic acid for contrast. The differentiated slides were washed in water, 75% ethanol, 95% ethanol, 100% ethanol, and fresh xylene, followed by mounting in mounting media (AGAR Scientific, Stansted, UK). The Giemsa-stained cremaster muscles were observed using a Leica DMRB upright microscope and a 25/0,75 NA oil immersion objective (both Leica, Wetzlar, Germany). Interstitial leukocytes were counted and differentiated into neutrophils, eosinophils, and mononuclear cells.

### 2.6. Preparation of Murine Aortic Endothelial Cells (MAECs)

The MAECs were isolated and cultured as previously described [31]. In brief, 3 mm long freshly harvested and cleaned aortic rings were seeded into Matrigel-coated culture dishes (BD, San Jose, CA, USA) and incubated at 37°C, 5% CO_2_ in Dulbecco's Modified Eagle Medium (supplemented with 15% fetal bovine serum, 1% Pen/Strep, 90 *μ*g/mL heparin, 60 *μ*g/mL endothelial cell growth supplement, and 1 *μ*g/mL amphotericin B; Fungizone, Invitrogen, Karlsruhe, Germany). After sufficient growth, endothelial cells were passaged with Dispase (BD, San Jose, CA, USA) and characterized by immunocytochemistry as described. For LPS-stimulation cells were grown in Costar 6-well plates (Corning Inc., Amsterdam, Netherlands) and standard medium to near confluence and incubated with LPS (*Escherichia coli*; serotype 055:B5 Sigma, Taufkirchen, Germany) at 100 ng/10^6^ cells for 3 hours at 37°C. Respective lipid pretreatment (Clinoleic, Lipofundin or Smoflipid at 10 mg/10^6^ cells) was initiated together with LPS-stimulation.

### 2.7. Isolation of Bone Marrow Neutrophils

Murine bone marrow PMNs were isolated from femurs and tibias as described previously [32]. After isolation, they were loaded on top of a discontinuous Percoll gradient (52%/64%/72%) and centrifuged at 1000 g for 30 minutes. PMNs were harvested from the 64%/72% interface and washed in PBS. PMN viability was greater than 95% as assessed by the trypan blue exclusion test, and purity was greater than 98% as analyzed by microscopy using Hemacolor staining (Merck, Darmstadt, Germany).

### 2.8. Flow Cytometry

For flow cytometric analysis of ICAM-1 and VCAM-1 expression on endothelial cells, prepared MAECs were harvested and incubated in the dark for 45 minutes on ice with PE-conjugated anti-ICAM-1 mAB (clone YN1/1.7.4 eBioscience, San Diego, CA, USA), anti-mouse VCAM-1 mAb (clone 429 MVCAM.A BioLegend, San Diego, CA, USA), or respective isotype control antibody (eBioscience, San Diego, CA, USA and BD) to detect anti-ICAM-1 and –VCAM-1 signal on 10.000 cells using the 4-decade FACS-Scan LSRII with DIVA software package (BD).

The expression of CXCR2, PSGL1, MAC1, and LFA1 was assessed using isolated bone marrow-derived neutrophils (see above). After red blood cell lysis, 10^6^ leukocytes/mL were stimulated for 3 h with 10 mg Lipofundin, Clinoleic, or Smoflipid, respectively, at 37°C. Next, cells were incubated in the dark with phycoerythrin-conjugated anti-CXCR2 mAb (1 *μ*g/10^6^ cells; eBioscience, Frankfurt, Germany), anti-PSGL-1 mAB (1 *μ*g/10^6^ cells, BD Pharmingen, San Diego, CA, USA), FITC-conjugated anti-MAC1 mAb M1/70 (1 *μ*g/10^5^ cells, rat IgG2b; eBioscience, San Diego, CA, USA), FITC-conjugated anti-LFA1 mAb M17/4 (1 *μ*g/10^5^ cells, rat IgG2a; eBioscience, San Diego, CA, USA), or respective isotype control antibodies (1 *μ*g/10^5^ cells, rat IgG2b or rat IgG2a; eBioscience, San Diego, CA, USA). The respective antigen expression was assessed on 10.000 cells per mouse within the neutrophil cluster defined by forward-side scatter analysis. Expression upon stimulation with different lipid emulsions was compared to unstimulated cells and their respective isotype controls. In certain experiments LPS was used for proinflammatory stimulation as indicated.

### 2.9. Flow Chamber Assay

Flow chamber experiments were conducted as described [33, 34]. In brief, rectangular microglass capillaries (VitroCom, Mountain Lakes, NJ, USA) were coated with rmP-selectin (2 *μ*g/mL), rmCXCL1 (5 *μ*g/mL), and ICAM-11 (1 *μ*g/mL) and connected via PE tubing to a 2 mL syringe containing freshly isolated bone marrow neutrophils from* LysEGFP *mice. In* LysEGFP *mice, the enhanced GFP (EGFP) is knocked into the murine lysozyme M (*lys*) locus leading to the expression of EGFP in myelomonocytic cells. The cell suspension (0.25 × 10^6^ GFP positive cells) was incubated with LPS (*Escherichia coli*; serotype 055:B5 Sigma, Taufkirchen, Germany, 100 ng/10^6^ cells for 3 hours at 37°C) and perfused through the flow chamber. Adhesion of GFP-positive cells was observed by fluorescence microscopy (BX51 WI with a saline immersion objective × 20/0.95 NA, Olympus Hamburg, Germany) for 10 minutes under constant flow conditions using a high precision perfusion pump (Harvard Instruments, March-Hugstetten, Germany; wall shear stress 0,1 Pa). Images were recorded via a CCD camera system (CF8HS; Kappa, Gleichen, Germany) on a Panasonic S-VHS recorder. In some experiments, cell suspensions were incubated with either Lipofundin, Smoflipid, or Clinoleic with a dose of 10 mg/10^6^ cells for 3 h at 37°C.

### 2.10. Model of Lethal Endotoxemia

Lethal endotoxemia was induced by a single i.p. injection of 40 mg/kg LPS (*Escherichia coli*; serotype 055:B5 Sigma, Taufkirchen, Germany) which was reconstituted in 100 *μ*L of sterile PBS, as reported previously [35]. Clinoleic, Smoflipid, Lipofundin, or equivalent volume of normal saline was administered i.v. at 2 g/kg 30 minutes, 8, and 24 hours after LPS challenge. In a first group, survival was observed for 14 days. In a second group, mice were perfused with saline and lungs were harvested 24 h after LPS injection. After fixation in PFA (4%) they were prepared for paraffin-embedded sections. Sections were performed at 3 *μ*m thickness and finally stained with H&E (haematoxylin and eosin staining) for microscopic evaluation.

### 2.11. Statistics

Sigma Stat 3.5 (Systat Software, Erkrath, Germany) was used for statistical analysis. Leukocyte counts, vessel diameters, leukocyte adhesion, leukocyte rolling flux fractions, wall shear rates, and in vitro leukocyte adhesion between groups and treatments were compared with one-way ANOVA followed by a multiple pairwise comparison test (Dunn's test) or by Wilcoxon rank-sum test, as appropriate. To compare the survival during lethal endotoxemia, log-rank test of Kaplan-Meier survival distribution was used. Statistical significance was set at *P* < 0.05.

## 3. Results and Discussion

### 3.1. Impact of Lipids on Leukocyte Recruitment during Trauma-Induced Cremaster Muscle Inflammation

Surgical preparation of the cremaster muscle induces leukocyte adhesion mainly via the chemokine CXCL1-CXCR2 pathway and *β*_2_ integrins LFA1 and MAC1 in the short-term model of trauma-induced inflammation [33, 34]. In our present experiments, we analyzed the number of adherent and rolling leukocytes in postcapillary venules of the mouse cremaster muscle in response to intravenous injection of Clinoleic, Smoflipid, Lipofundin, or normal saline. To confirm the systemic availability of the injected lipid, we first showed that blood triglyceride levels significantly increased compared to controls after all three lipids, while blood levels of cholesterol and standard liver enzymes stayed unaltered (see Supplemental Table 1 in Supplementary Material available online at http://dx.doi.org/10.1155/2015/757059). Notably, the varying composition of investigated lipid emulsions did not lead to significant differences in blood triglyceride levels. Next, we ruled out that alterations of leukocyte recruitment might be caused by hemodynamic changes after fluid injection, since there were no differences in hemodynamic and microvascular parameters between the different treatment groups (Supplemental Table 2).

**Figure 1:**
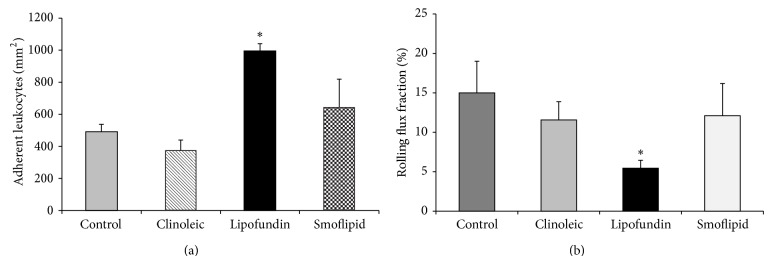
Effect of different lipid compositions on leukocyte recruitment in the trauma model. Leukocyte adhesion (a) as number of adherent cells per mm^2^ of surface area and rolling (b) as rolling flux fraction in cremaster muscle venules of mice treated with Clinoleic, Lipofundin, Smoflipid (1 g/kg), or normal saline (control) during trauma-induced inflammation were investigated using intravital microscopy. All values are presented as mean ± SEM from three or more mice per group. Significant differences (*P* < 0.05) to control mice are indicated by the asterisk.

After injection of 1 g/kg Lipofundin, the number of adherent leukocytes significantly increased when compared to control conditions (Figure 1(a)). While the same amount of Smoflipid slightly increased leukocyte adhesion, Clinoleic injection resulted in an insignificant decrease of adherent leukocytes. Since Lipofundin significantly reduced the number of rolling leukocytes (rolling flux fraction) when compared to controls (Figure 1(b)), its proinflammatory stimulation triggers the transition from leukocyte rolling to adhesion and rolling leukocytes adhere more frequently. Neither Clinoleic nor Smoflipid treatment altered leukocyte rolling compared to controls.

As reported previously during that mild and short-term inflammation of the trauma model, anti-inflammatory effects of candidate substances are less common than proinflammatory effects [29, 30]. Therefore, we argue that potentially anti-inflammatory lipid effects are difficult to examine in that model, although there was an obvious proinflammatory status in response to Lipofundin. Therefore, we continued with another established inflammation model of the mouse cremaster muscle.

### 3.2. Impact of Lipids on Leukocyte Adhesion during LPS-Induced Cremaster Muscle Inflammation

As a potent proinflammatory agent, we administered LPS in a dose of 50 ng intrascrotally 3 h prior to exteriorization of the cremaster muscle and observed the lipid-induced effects on leukocyte adhesion in murine cremaster muscle venules.

Microvascular and hemodynamic parameters were similar between the investigated groups (Supplemental Table 3).

**Figure 2:**
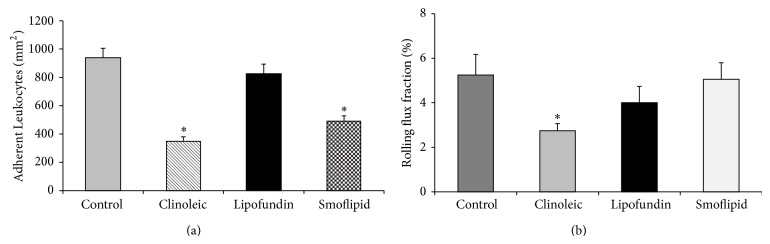
Effect of different lipid compositions on leukocyte recruitment during LPS-induced inflammation. Leukocyte adhesion (a) as number of adherent cells per mm^2^ of surface area and rolling (b) as rolling flux fraction in cremaster muscle venules of mice treated with Clinoleic, Lipofundin, Smoflipid (1 g/kg), or saline (control) were investigated by intravital microscopy 3 h after intrascrotal administration of LPS (50 ng/mouse). Lipids were administered simultaneously with LPS. All values are presented as mean ± SEM from three or more mice per group. Significant differences (*P* < 0.05) to saline-treated mice are indicated by the asterisk.

In the model of LPS-induced inflammation, a proinflammatory status is induced by TNF*α*-mediated upregulation of chemokines and adhesion molecules on leukocytes and the endothelium [34, 36, 37]. This strong inflammation after intrascrotal injection of LPS is reflected by profound leukocyte adhesion in control mice (Figure 2(a)). The LPS-induced leukocyte adhesion was robustly blocked by Clinoleic and less pronounced by Smoflipid. In contrast, administration of Lipofundin did not alter leukocyte adhesion in this model when compared to control mice (Figure 2(a)). As depicted in Figure 2(b), leukocyte rolling was not affected by Lipofundin and Smoflipid, whereas rolling flux fraction was significantly reduced by Clinoleic treatment when compared to controls. Therefore, we suggest that Clinoleic is able to interfere with both leukocyte rolling and adhesion. We next analyzed the number of transmigrated leukocytes in cremaster muscle whole mounts in the respective treatment groups, postulating that the lipid induced inhibition of leukocyte adhesion largely translates into transmigration (Supplemental Figure 1). Notably, Lipofundin seems to affect neutrophil transmigration more than leukocyte adhesion.

The protective effects of Smoflipid and Clinoleic on leukocyte recruitment are most likely explained by their specific olive oil and/or fish oil composition [17]. In line with our study, Glatzle et al. found a decrease of LPS- (5 mg/kg i.p.) induced leukocyte recruitment after treatment with Clinoleic and less pronounced also with Smoflipid. However, lipids were applied enterally in their study which is often not feasible in patients in the intensive care unit [17].

Our observations seem to contrast the study of Buenestado et al. in rat mesenterium, which described a Lipofundin-induced inhibition of the whole leukocyte recruitment cascade after superfusion with LPS but no such effect in response to Clinoleic [16]. The conflicting results, however, might be due to different experimental setups (LPS application, lipid administration, and different investigated tissues and species) leading to different involved signaling pathways.

As a summary of our intravital microscopic experiments, we found that among all investigated lipids Clinoleic blocked leukocyte recruitment most strongly, indicating a protective role of olive oil (omega-9 fatty acids) during inflammation.

### 3.3. Anti-Inflammatory Properties of Lipids during Lethal Endotoxemia

Next, we aimed to investigate immunomodulatory effects of lipids in a clinically more relevant and well-established mouse model of lethal endotoxemia. In this inflammatory model, an intraperitoneal injection of* Escherichia coli* LPS (40 mg/kg) is followed by treatment with 2 g/kg of the respective lipids (Clinoleic, Smoflipid, or Lipofundin) or control solution (equivalent volume of normal saline) after 0.5, 8, and 24 hours.

**Figure 3:**
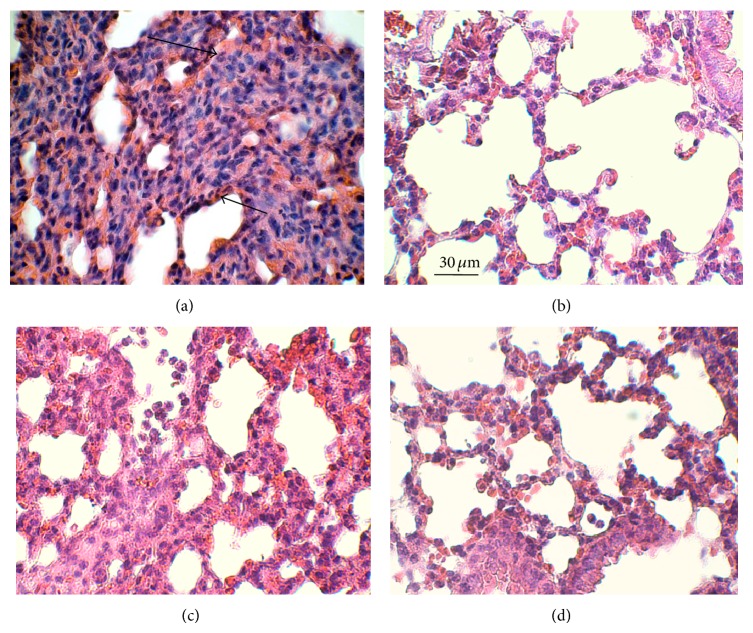
Effect of treatment with Lipofundin, Smoflipid, or Clinoleic on lung inflammation during LPS-induced endotoxemia. Lethal endotoxemia was induced by* Escherichia coli *LPS (serotype 055:B5, 40 mg/kg i.p.) and treated with 2 g/kg Clinoleic (b), Lipofundin (c), Smoflipid (d), or normal saline (control, (a)) at 0.5, 8, and 24 h after LPS challenge. Lungs were harvested 24 h after LPS challenge and prepared as 3 *μ*m paraffin-embedded sections for H&E staining. Representative micrographs are shown for at least three mice per group. Arrows indicate infiltrating neutrophils. Reference bar for (a)–(d) is shown in (b) and represents 30 *μ*m.

To quantify organ infiltration, some mice were used to investigate leukocyte infiltration into the lung 24 h after LPS-injection. We observed an increased leukocyte number after application of LPS (Figure 3(a)) that was unchanged after injection of Lipofundin (Figure 3(c)) and hardly improved after injection of Smoflipid (Figure 3(d)). In line with our results in the above-mentioned inflammation models, we found a marked reduction of infiltrated PMN after application of Clinoleic (Figure 3(b)). Garnacho-Montero et al. hypothesized that protective effects of Clinoleic during pulmonary inflammation may be caused by its antioxidative properties [38].

**Figure 4:**
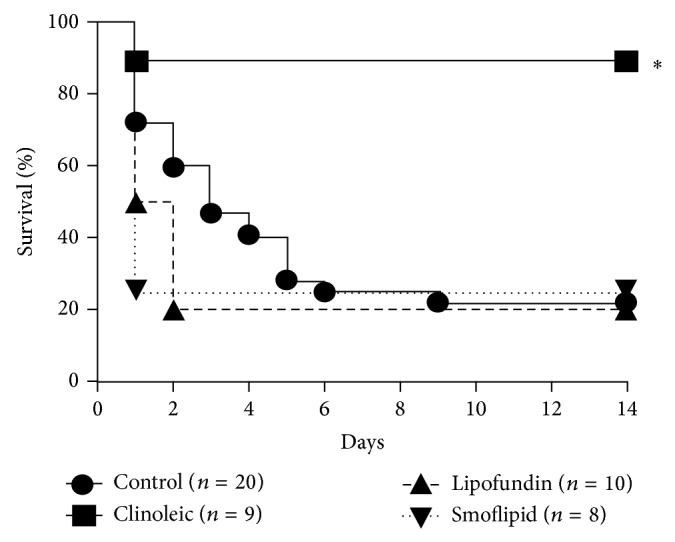
Lipid-dependent survival during LPS-induced endotoxemia. Lethal endotoxemia was induced by* Escherichia coli *LPS (serotype 055:B5, 40 mg/kg i.p.) and treated with either 2 g/kg Lipofundin, Smoflipid, Clinoleic, or normal saline (control) at 0.5, 8, and 24 h i.v. after LPS challenge. Survival is shown in Kaplan-Meier plots for the respective treatments. Significant differences by log-rank test were set at *P* < 0.05 and indicated by the asterisk.

In a second group, survival was observed for 14 days. Consistent with previous findings, survival of control mice was about 20% [29] which stayed unaffected after injection of Lipofundin and Smoflipid (20% and 25%, resp., Figure 4). In contrast, Clinoleic strongly improved survival during lethal endotoxemia when compared to controls (90% versus 20%, resp.).

These results are consistent with former studies that observed a protective effect of olive oil in septic mice [39] and critically ill patients [17, 40]. However, they are in contrast to other studies describing an anti-inflammatory function of Smoflipid during endotoxemia [41–43]. Moreover, Boisramé-Helms et al. investigated membrane remodeling during peritonitis-induced septic shock in rats and found proinflammatory effects of MCT/LCT but not of LCT only [5]. This is in line with our findings, since Clinoleic only contains LCT (besides olive oil). Therefore, the varying triglyceride composition of the investigated lipid emulsions might be another reason for their different anti-inflammatory effects. Taken together, in critically ill patients the optimal lipid substitution is still not clear [4].

### 3.4. Lipid Dependent Leukocyte Adhesion during Flow Chamber Experiments

**Figure 5:**
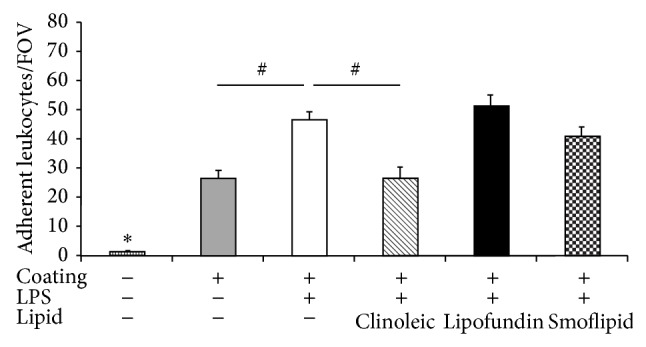
Effect of different lipid treatments on LPS-induced leukocyte adhesion in flow chamber experiments. Microflow chambers were coated with P-selectin, ICAM-1, and CXCL1 and perfused with LPS-stimulated bone marrow neutrophils at constant flow. Leukocyte adhesion (adherent leukocytes per field of view) was assessed after pretreatment with or without Clinoleic, Lipofundin, or Smoflipid and compared to unstimulated and uncoated control flow chambers. Significant differences versus all other groups are indicated by the asterisk and between indicated groups by the pound key (*P* < 0.05).

Next, we aimed to dissect leukocyte from endothelium driven mechanisms mediating lipid-induced inhibition of leukocyte adhesion and performed flow chamber experiments. Therefore, microflow chambers were coated with P-selectin, ICAM-1, and CXCL1 and constantly perfused with LPS-stimulated isolated murine bone marrow neutrophils with and without lipid pretreatment. We observed a significant number of adherent leukocytes in coated flow chambers when compared to uncoated flow chambers (Figure 5). Noteworthy, LPS stimulation further augmented leukocyte adhesion. While Clinoleic significantly blocked LPS-induced leukocyte adhesion, Lipofundin or Smoflipid did not show such effect. These results indicate that (amongst others) leukocyte-born mechanisms are likely to be connected to Clinoleic-induced inhibition of leukocyte recruitment, whereas any immunomodulatory effects of Lipofundin or Smoflipid might rather be linked to other mechanisms. Altered cytokine and chemokine release could play a role in this context [38, 44].

### 3.5. Impact of Lipids on Expression of Leukocyte Adhesion Molecules on Bone Marrow Derived Neutrophils

**Figure 6:**
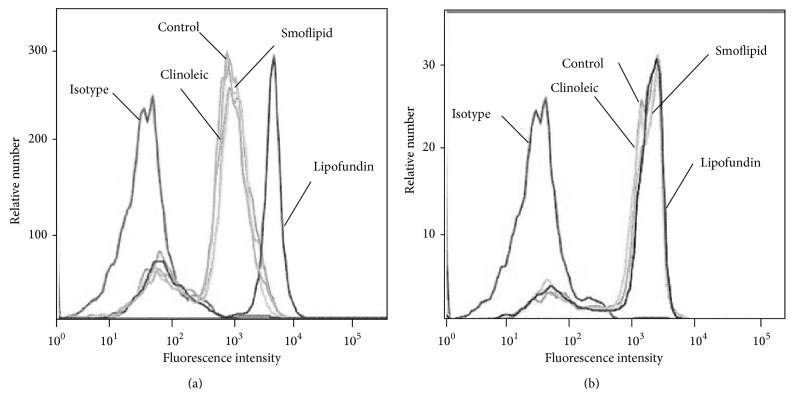
Lipid-dependent expression of LFA1 and MAC1. Surface expression of MAC1 (a) and LFA1 (b) on bone marrow-derived neutrophils (*n* = 3 mice) was compared to untreated controls after incubation with Lipofundin, Smoflipid, or Clinoleic (10 mg per 10^6^ leukocytes/mL, 3 h at 37°C). Representative histograms are shown from 3 separate experiments.

To further explore underlying mechanisms for the observed lipid-induced effects, we analyzed the expression of the *β*_2_ integrins LFA1 and MAC1 on neutrophils in response to lipid incubation. Lipofundin induced an upregulation of MAC1, while neither Smoflipid nor Clinoleic had any effects on the expression of MAC1 (Figure 6(a)). As depicted in Figure 6(b), expression of LFA1 on bone marrow derived mouse neutrophils was not altered after any lipid incubation when compared to control neutrophils. This finding emphasizes a proinflammatory effect of Lipofundin and is in line with Versleijen et al. demonstrating that neutrophil activation can be triggered by MCT/LCT [45]. In contrast, the study of Buenestado et al. [16] did not observe any effects of Lipofundin (MCT/LCT) on integrin expression. The conflicting results might be attributable to a different experimental setting and a shorter incubation period. Nevertheless, our results also indicate that the anti-inflammatory effect of Clinoleic is not based on downregulation of *β*_2_ integrins.

**Figure 7:**
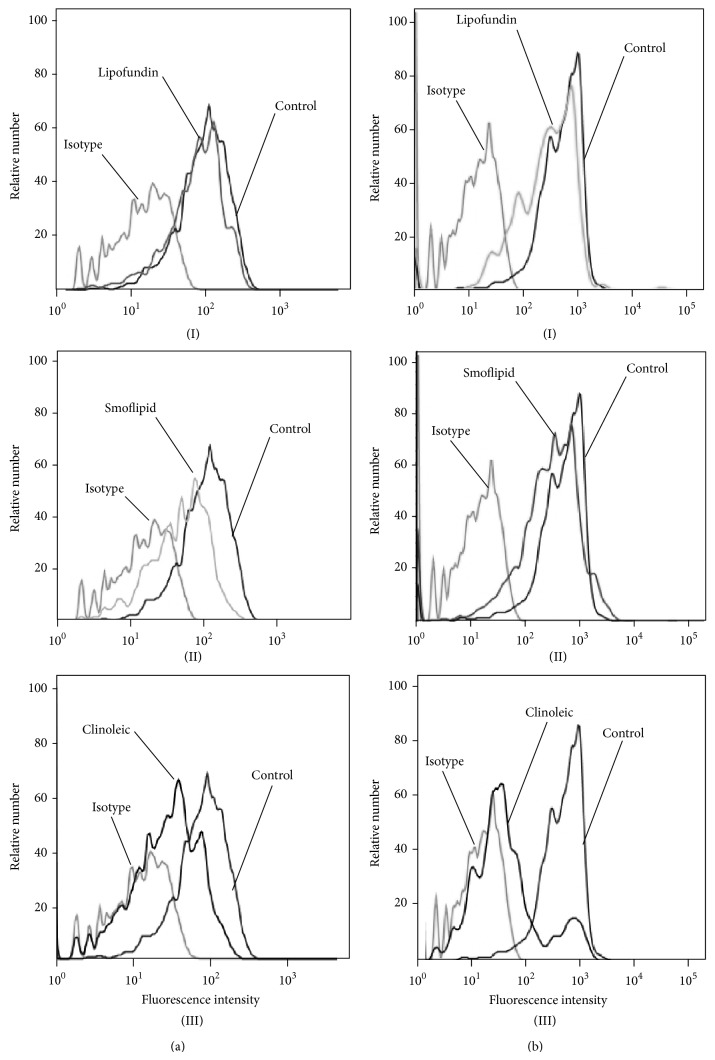
Lipid-dependent expression of CXCR2 and PSGL1. Surface expression of CXCR2 (a) and PSGL1 (b) on bone marrow-derived neutrophils (*n* = 3 mice) was compared to untreated controls after incubation with Lipofundin (I), Smoflipid (II), or Clinoleic (III) (10 mg per 10^6^ leukocytes/mL, 3 h at 37°C). Representative histograms are shown from 3 separate experiments.

Thus, we next investigated the influence of the respective lipid emulsions on the expression of PSGL1 and CXCR2. PSGL1 is expressed on neutrophil granulocytes and mediates their recruitment to inflamed tissues via binding to selectins [46]. CXCR2 expression was downregulated by both Clinoleic and Smoflipid, but not by Lipofundin (Figure 7(a)). PSGL1 showed a marked downregulation after application of Clinoleic only, whereas Smoflipid and Lipofundin displayed no effect (Figure 7(b)). These results suggest that anti-inflammatory properties of Clinoleic are mediated by PSGL1 and CXCR2 and those of Smoflipid by CXCR2, which is in line with our observation of reduced LPS-induced leukocyte rolling after Clinoleic administration only. Although Versleijen et al. demonstrated that neutrophil activation can be triggered by MCT/LCT, this does not seem to involve leukocyte-expressed PSGL1 or CXCR2 [45].

### 3.6. Impact of Lipids on Endothelial Expression of Leukocyte Adhesion Molecules

**Figure 8:**
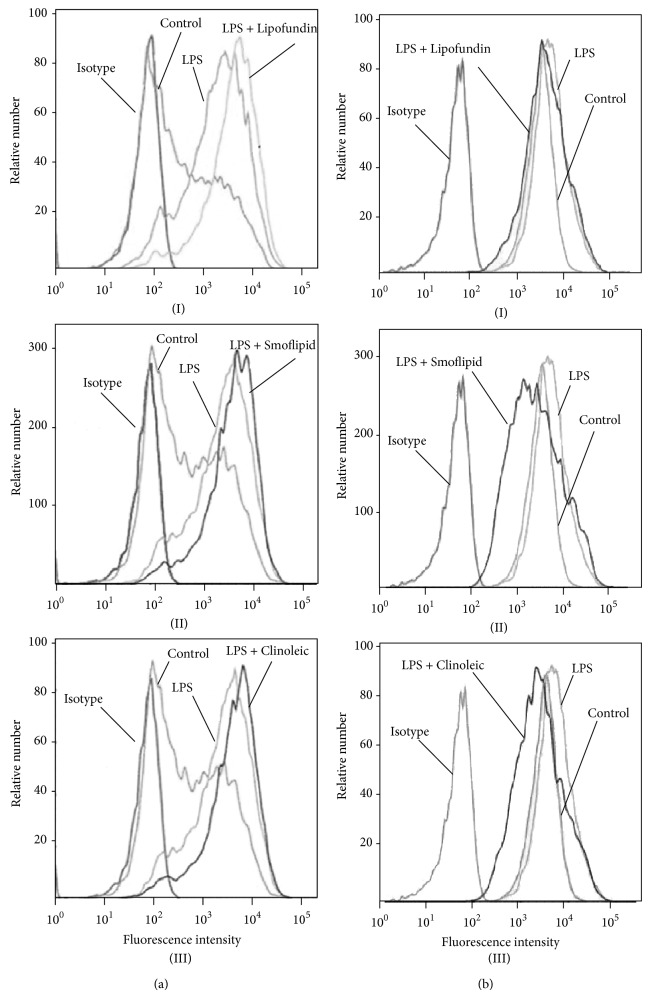
Lipid-dependent endothelial ICAM-1 and VCAM-1 expression. ICAM-1 (a) and VCAM-1 (b) expression of cultured endothelial cells was measured after stimulation with LPS (100 ng/10^6^ leukocytes/mL) and simultaneous treatment with Lipofundin (I), Smoflipid (II), or Clinoleic (III) (10 mg per 10^6^ leukocytes/mL, 3 h at 37°C). Endothelial ICAM-1 and VCAM-1 expressions were compared to respective isotype and untreated (LPS) and unstimulated (control) controls. Representative histograms are shown for three separate experiments.

We next addressed the question whether lipids alter the expression of endothelial leukocyte adhesion molecules like ICAM-1 and VCAM-1. Therefore, LPS-stimulated ICAM-1 and VCAM-1 expression was assessed on MAECs by flow cytometry. In line with previous studies, LPS induced an upregulation of ICAM-1 and—less pronounced—of VCAM-1. While none of the applied lipids altered ICAM-1-expression (Figure 8(a)), LPS-induced VCAM-1 expression was downregulated by Smoflipid and Clinoleic (Figure 8(b)), indicating that an anti-inflammatory effect of omega 3 and omega 9 fatty acids might be attributable to endothelial VCAM-1 downregulation in our experimental setting. This finding is supported by the study of Tull et al. which investigated endothelial mechanisms of lipid-mediated immunomodulation [44].

## 4. Conclusion

Clinoleic exerted the strongest anti-inflammatory properties during local and systemic inflammation in vivo when compared to the lipid composition Smoflipid or Lipofundin. In turn, Clinoleic was the only investigated lipid emulsion that improved survival during lethal endotoxemia. Although olive oil-based lipids seem to be a beneficial alternative to other lipid emulsions, future studies are needed to confirm the anti-inflammatory potential of Clinoleic in critically ill patients.

## Supplementary Material

Supplemental Table 1. Murine blood serum levels of lipids and liver enzymes after lipid treatment.
Supplemental Table 2. Hemodynamic and microvascular parameters of cremaster muscle venules after lipid treatment during trauma-induced inflammation.
Supplemental Table 3. Hemodynamic and microvascular parameters of cremaster muscle venules after lipid treatment during LPS-induced inflammation.
Supplemental Figure 1. Effect of different lipid compositions on neutrophil infiltration into LPS-stimulated cremaster msucles.
