# Supplementary material for: The Olive Oil-Based Lipid Clinoleic Blocks Leukocyte Recruitment and Improves Survival during Systemic Inflammation: A Comparative In Vivo Study of Different Parenteral Lipid Emulsions
Source: Mediators Inflamm. 2015 Feb 12;2015:757059. doi: 10.1155/2015/757059 (PMC4341856; doi:10.1155/2015/757059)

SUPPLEMENTAL MATERIAL

Supplemental Table 1. Murine blood serum levels of lipids and liver enzymes after lipid treatment. Triglycerides, cholesterol, glutamic oxaloacetic transaminase (GOT) and glutamic pyruvic transaminase (GPT) are displayed 3h after intravenous administration of 1 g/kg of different lipid emulsions or normal saline. Data are presented as mean ± SEM; n.s. = not significant.

	Mice N	*Triglycerides* (mg/dl)	*Cholesterol* (mg/dl)	*GOT* (U/l)	*GPT* (U/l)
Control	6	50 ± 10	100 ± 10	230 ± 40	100 ± 30
Clinoleic	9	130 ± 20*	90 ± 10	190 ± 30	80 ± 10
Smoflipid	9	90 ± 10*	80 ± 10	190 ± 30	75 ± 10
Lipofundin	7	90 ± 10*	90 ± 10	250 ± 30	110 ± 15
		* vs. control	n.s.	n.s.	n.s.

.

Supplemental Table 2. Hemodynamic and microvascular parameters of cremaster muscle venules after lipid treatment during trauma-induced inflammation. Vessel diameter, centerline velocity, and wall shear rate are displayed after intravenous administration of 1g/kg of different lipid emulsions or normal saline. Data are presented as mean ± SEM; n.s. = not significant.

	Mice	*Venules*	*Diameter*	*Centerline Velocity*	*Wall Shear Rate*	*Systemic Leukocyte Counts*
	N	n	(µm)	(µm/s)	(s-1)	(/µl)
Control	4	18	28 ± 1	2200 ± 100	1900 ± 100	6300 ± 100
Smoflipid	4	12	28 ± 1	2200 ± 50	2200 ± 150	5100 ± 600
Clinoleic	4	18	28 ± 1	2200 ± 50	2000 ± 100	7300 ± 600
Lipofundin	3	29	27 ± 1	2100 ± 50	2000 ± 100	4900 ± 500
			n.s.	n.s.	n.s.	n.s.

Supplemental Table 3. Hemodynamic and microvascular parameters of cremaster muscle venules after lipid treatment during LPS-induced inflammation. Vessel diameter, centerline velocity, and wall shear rate are displayed after intravenous administration of 1g/kg of different lipid emulsions or normal saline. Data are presented as mean ± SEM; n.s. = not significant.

	Mice	*Venules*	*Diameter*	*Centerline Velocity*	*Wall Shear Rate*	*Systemic Leukocyte Counts*
	N	n	(µm)	(µm/s)	(s-1)	(/µl)
Control	3	21	27 ± 1	2100 ± 50	1900 ± 100	3700 ± 100
Clinoleic	3	34	26 ± 1	2100 ± 50	2100 ± 100	4400 ± 200
Smoflipid	3	34	27 ± 1	2100 ± 50	1900 ± 100	6200 ± 500
Lipofundin	3	29	26 ± 1	2100 ± 50	2000 ± 100	6500 ± 200
			n.s.	n.s.	n.s.	n.s.

Supplemental Figure 1. Effect of different lipid compositions on neutrophil infiltration into LPS-stimulated cremaster msucles. Neutrophil infiltration (number of transmigrated neutrophils per mm^2 of perivascular surface area) in Giemsa-stained cremaster muscle whole mounts of mice treated with Clinoleic, Lipofundin, Smoflipid (1g/kg) or saline (control) was investigated after intravital microscopy in the LPS model. All values are presented as mean ± SEM from three or more mice per group. Significant differences ($p < 0.05$) to saline treated mice are indicated by the asterisk.

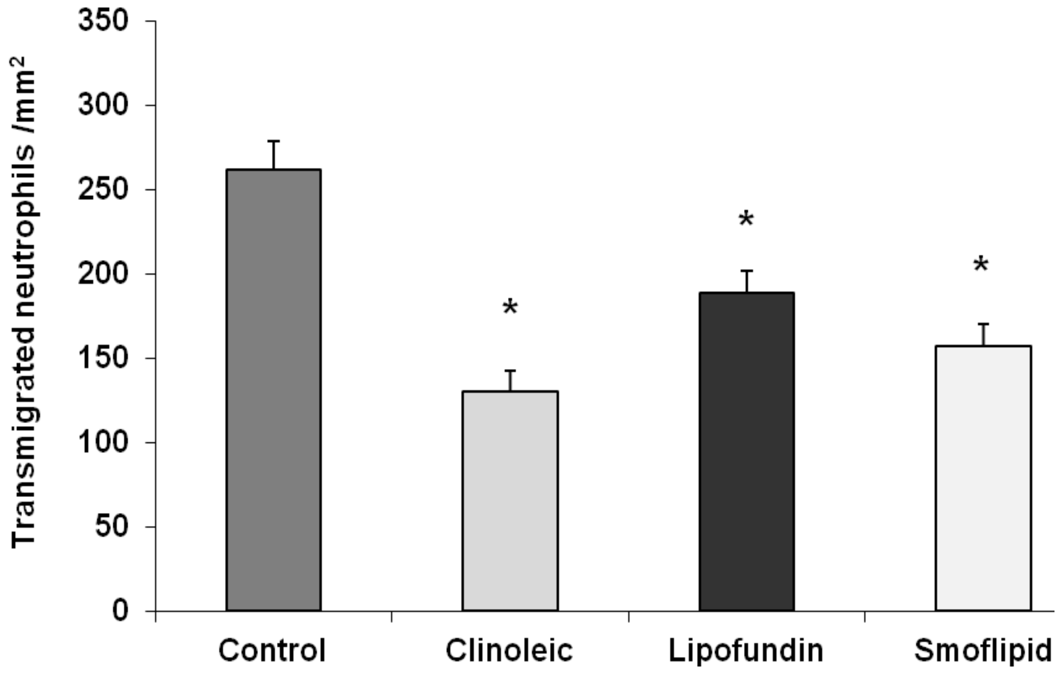

Supplement: Supplementary file 1 — Supplemental Table 1. Murine blood serum levels of lipids and liver enzymes after lipid treatment. Supplemental Table 2. Hemodynamic and microvascular parameters of cremaster muscle venules after lipid treatment during trauma-induced inflammation. Supplemental Table 3. Hemodynamic and microvascular parameters of cremaster muscle venules after lipid treatment during LPS-induced inflammation. Supplemental Figure 1. Effect of different lipid compositions on neutrophil infiltration into LPS-stimulated cremaster msucles. [file 757059.f1.pdf]
